# Genomic analyses based on pulmonary adenocarcinoma in situ reveal early lung cancer signature

**DOI:** 10.1186/s12920-018-0413-3

**Published:** 2018-11-20

**Authors:** Dan Li, William Yang, Yifan Zhang, Jack Y Yang, Renchu Guan, Dong Xu, Mary Qu Yang

**Affiliations:** 1 Key Laboratory of Symbolic Computation and Knowledge Engineering of Ministry of Education, College of Computer Science & Technology, Jilin University, Changchun, 130012 China; 2 MidSouth Bioinformatics Center and Joint Bioinformatics Ph.D. Program of University of Arkansas at Little Rock and Univ. of Arkansas Medical Sciences, 2801 S. Univ. Ave, Little Rock, AR 72204 USA; 3 Department of Computer Science, Carnegie Mellon University School of Computer Science, 5000 Forbes Ave, Pittsburgh, PA 15213 USA; 4 Department of Electrical Engineering and Computer Science, Informatics Institute, and Christopher S. Bond Life Sciences Center, University of Missouri, Columbia, MO 65211 USA

**Keywords:** Adenocarcinoma in situ, AIS, Lung cancer, Invasive, Early diagnosis, lncRNAs

## Abstract

**Background:**

Non-small cell lung cancer (NSCLC) represents more than about 80% of the lung cancer. The early stages of NSCLC can be treated with complete resection with a good prognosis. However, most cases are detected at late stage of the disease. The average survival rate of the patients with invasive lung cancer is only about 4%. Adenocarcinoma in situ (AIS) is an intermediate subtype of lung adenocarcinoma that exhibits early stage growth patterns but can develop into invasion.

**Methods:**

In this study, we used RNA-seq data from normal, AIS, and invasive lung cancer tissues to identify a gene module that represents the distinguishing characteristics of AIS as AIS-specific genes. Two differential expression analysis algorithms were employed to identify the AIS-specific genes. Then, the subset of the best performed AIS-specific genes for the early lung cancer prediction were selected by random forest. Finally, the performances of the early lung cancer prediction were assessed using random forest, support vector machine (SVM) and artificial neural networks (ANNs) on four independent early lung cancer datasets including one tumor-educated blood platelets (TEPs) dataset.

**Results:**

Based on the differential expression analysis, 107 AIS-specific genes that consisted of 93 protein-coding genes and 14 long non-coding RNAs (lncRNAs) were identified. The significant functions associated with these genes include angiogenesis and ECM-receptor interaction, which are highly related to cancer development and contribute to the smoking-free lung cancers. Moreover, 12 of the AIS-specific lncRNAs are involved in lung cancer progression by potentially regulating the ECM-receptor interaction pathway. The feature selection by random forest identified 20 of the AIS-specific genes as early stage lung cancer signatures using the dataset obtained from The Cancer Genome Atlas (TCGA) lung adenocarcinoma samples. Of the 20 signatures, two were lncRNAs, BLACAT1 and CTD-2527I21.15 which have been reported to be associated with bladder cancer, colorectal cancer and breast cancer. In blind classification for three independent tissue sample datasets, these signature genes consistently yielded about 98% accuracy for distinguishing early stage lung cancer from normal cases. However, the prediction accuracy for the blood platelets samples was only 64.35% (sensitivity 78.1%, specificity 50.59%, and AUROC 0.747).

**Conclusions:**

The comparison of AIS with normal and invasive tumor revealed diseases-specific genes and offered new insights into the mechanism underlying AIS progression into an invasive tumor. These genes can also serve as the signatures for early diagnosis of lung cancer with high accuracy. The expression profile of gene signatures identified from tissue cancer samples yielded remarkable early cancer prediction for tissues samples, however, relatively lower accuracy for boold platelets samples.

**Electronic supplementary material:**

The online version of this article (10.1186/s12920-018-0413-3) contains supplementary material, which is available to authorized users.

## Background

Lung cancer is one of the most common cancer types and the main cause of cancer-related deaths. About 14% of all new cancers are lung cancers, and about 154,050 deaths from lung cancer are estimated in the United States for 2018 by the American Cancer Society. Non-small cell lung cancer accounts for about 80% of the lung cancer cases and is consist of various subtypes [1]. Generally, most of the deaths caused by lung cancer are in late stages which are due to the distant metastasis and invasion [2]. In contrast, the early stages or non-invasive subtypes of lung cancer can be cured [2].

Lung adenocarcinoma in situ is a subtype of NSCLC and shows non-invasive growth patterns. The 5-year survival rate of AIS is almost 100% with appropriate therapy [3]. However, AIS can develop into an invasive stage of lung cancer that has only approximate 4% patient survival rate [1]. AIS is different from the other lung cancer histologies in that most AIS patients are non-smokers and women [4, 5]. Previous studies of AIS, for purposes of classification and diagnosis, have indicated differences in appearance from these and other types of lung cancer. The studies of AIS at the genetic level have not yet been widely performed, consequenctly, our understanding of the mechanism that causes AIS is limited. On the other hand, AIS cases could be missed diagnosed as pneumonia since sometimes AIS has a varied appearance on CT [6] and generally 62% of the AIS patients do not have symptoms [7]. Similarly, early stage lung cancer often is asymptomatic.

Previous studies have identified gene biomarkers involved in lung cancer progression and development [8], including several critical long non-coding RNAs [2, 9, 10]. More effective and robust molecular biomarkers for early lung cancer diagnosis remained to be uncovered. Currently, studies on AIS progression based on RNA sequencing techniques were performed. Some protein-coding genes and lncRNAs that related to AIS were identified [3] and indicated the evolution of lung cancer from normal to invasive stages. However, large-scale study and comparison of these genes at different disease stage of cancer development are not exploited.

In this study, we first identified the genes that were specifically expressed in AIS tissue samples compared with normal and invasive cancer cases simultaneously. The differential expression analysis was performed by using two computational methods, the most widely used edgeR [11] and the newly developed Cross-Value Association Analysis (CVAA) [12]. The combined results of these two methods were used for downstream analysis. Only a small group of genes (107) including both protein-coding genes (94) and lncRNAs (13) were found that potentially dominate the AIS and the invasive progression (Additional file 1: Figure S1). Smoking is considered one of the most risk factors that cause lung cancers and about 75% of the lung cancer cases are attributable to tobacco use. The lung cancer in never smokers even considered as different diseases [5]. The AIS-specific genes were significantly enriched of lung cancer related functional annotations such as angiogenesis [13, 14] and the ECM-receptor interaction which is a known pathway contributes the smoke-free lung cancers [15–17]. We further identified 20 early lung cancer signature genes that can be used for distinguishing the early lung cancer cases from normal ones. In particular, we performed an experiment using the random forest method on four independent datasets generated by RNA-seq or microarray techniques and achieved about 98% prediction accuracy for early stage lung cancer in tissue samples but only 64.35% overall accuracy in the blood platelets dataset.

Our results suggested that AIS-specific genes could help us to better understand this uncommon lung cancer subtype. The AIS-specfic genes may also play a critical role in the lung cancer progression. Moreover, the expression profiles of early lung cancer signature genes we identified showed the ability for accurate and robust early cancer prediction.

## Results

### Comparison of gene expression in AIS and invasive lung cancer

To investigate the genes that dominate the intermediate type of AIS and underlie different phenotypes (normal, AIS and invasive cancer cases), we collected the RNA-seq library (GSE52248) consisted of normal, AIS and invasive cancer samples of six lung cancer patients [3]. The raw RNA-seq data were generated from formalin fixation and paraffin embedding (FFPE) processed tissues. First, the RNA-seq data were processed and the gene expression profile was calculated referring the gene annotation from Ensembl (Methods). Then, the differential expression analysis via edgeR was performed on 16,501 expressed genes consisted of 15,106 protein-coding genes and 1395 lncRNAs. As a result, 1348 significant differentially expressed genes (DEGs) were found between normal and invasive lung cancer samples under the threshold |log2 fold change| > 1 & FDR < 0.05. Based on the same thresholds, 719 DEGs between normal and AIS cases as well as 98 DEGs between AIS and invasive cancer tissues were identified. The gene expression patterns in AIS and invasive cancer tissues demonstrated much more consistency (Additional file 1: Figure S1) despite these two phenotypes was with great differences. Our results indicated that only a small number of genes potentially dominated the evolution of lung cancer from AIS into invasive lung cancer.

### Identification of AIS-specific genes

To comprehensively identify the gene set that was specifically expressed in AIS tissue, we applied two differential expression analysis methods, edgeR [11] and CVAA [12], based on the gene expression profiles of paired normal and AIS, AIS and invasive cancer samples. The edgeR is one of the most widely used differential expression (DE) analysis method, while CVAA is a newly developed normalization-free and nonparametric DE analysis method. Unlike the commonly used DE analysis methods, CVAA neither normalizes nor assumes the distribution of the gene expressions. Instead, it reveals the DEGs according to the gene expression comparison and ranking. The DEGs between normal and AIS that, at the same time were differentially expressed in invasive cancer compared with AIS samples were further used as the candidates for AIS-specific genes (Methods). The union set of the DEGs identified by the two methods was collected. As a result, a total of 107 (22 upregulated and 85 downregulated) genes including 93 protein-coding genes and 14 long non-coding RNAs were identified as AIS-specific genes (Methods, Additional file 2: Table S1).

### LncRNAs potentially regulate ECM-receptor interaction pathway and involved in lung cancer

We applied the function annotation via David [18] on the 93 protein-coding genes and found a number of enriched functions (Additional file 3: Table S2), including angiogenesis and ECM-receptor interaction which shows the aggressiveness of the tumor and has an important role in metastasis [13, 14]. A previous study of lung cancer [17] indicated that non-smokers also have the risk of the lung cancer. Some well-known cancer-related pathways such as cell cycle and p53 were enriched of differentially expressed genes in only current smoke patients, whereas ECM-receptor interaction pathway is over-represented in the patients that never smoke and is considered to contribute to smoking-independent lung cancer [17]. Interestingly, it has been found that AIS is more common in women and non-smokers [3] and the disrupted ECM-receptor interaction pathway was also found based on the AIS data in our study. Many ECM proteins are factors that promote the metastatic cascade as they are significantly deregulated during the progression of cancer [16].

The ECM-receptor interaction pathway contains 87 protein-coding genes and three of them (*CD36, SPP1, TNR*) are AIS-specific. We further employed GENIE3 (Gene Network Inference with Ensemble of trees) [19] to predict the regulatory relationships between the 14 AIS-specific lncRNAs and the 87 genes (Methods). As a result, 12 lncRNAs were found to potentially regulate the genes in ECM-receptor interaction pathway (Additional file 4: Figure S2), suggesting their roles in the lung cancer progression. Moreover, the odd ratios of the regulations between the lncRNAs and the ECM-receptor interaction pathway indicated novel lncRNAs, such as FENDRR (OR = 1.53), MEOX2-AIS (OR = 3.22), as regulators interact with this pathway (Methods). Collectively, these results suggested that the AIS-specific genes played critical roles in the progression of AIS and the development of invasive lung cancer.

### Early lung cancer signatures identification

**Fig. 1:**
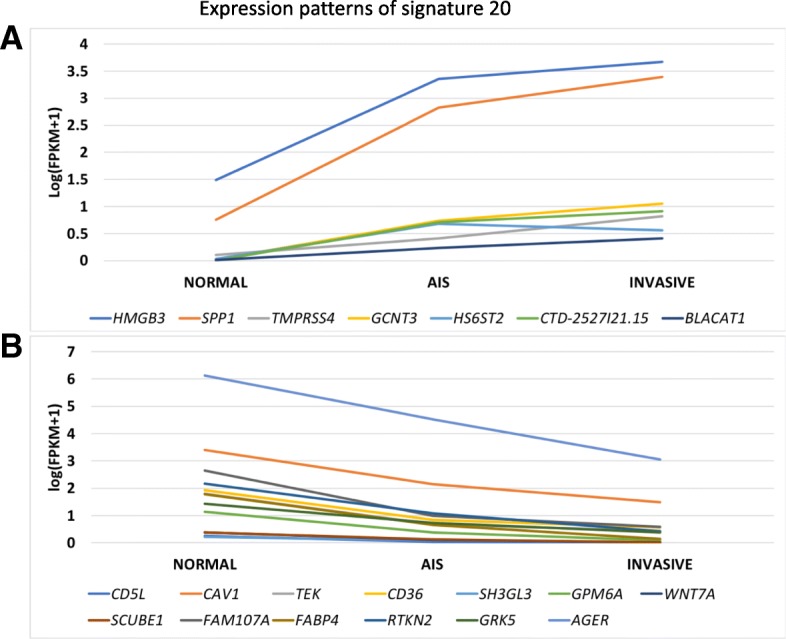
The gene expression patterns of the 20 early lung cancer signatures. A, seven genes including the two lncRNAs were upregulated along with the lung cancer progression from normal to invasive. B, 13 genes were continually downregulated

AIS is a pre-invasive lung adenocarcinoma lesion. Hence, the AIS-specific genes can potentially serve as gene signatures for early lung cancer detection. We employed random forest for selecting the top genes from the 107 AIS-specific genes that can effectively distinguish normal from early-stage cancer cases (Methods). Using the gene expression profiles of the normal (*n* = 59) and early-stage (stage I) lung adenocarcinoma cases (*n* = 286) from TCGA project, random forest reported the importance of each gene by calculating the classification error rate. We found that one gene set composed of 20 genes yielded the lowest error rate (1.16%). Therefore, these 20 genes including two lncRNAs (BLACAT1, CTD-2527I21.15) ranked by the importance scores of random forest were considered to be early lung cancer diagnosis signatures and were used for further validation and analysis (Additional file 5: Table S3). Of the 20 gene signatures, 13 were continually downregulated along with the lung cancer progression from normal to AIS to invasive. In contrast, the expression levels of the other seven genes were significantly increased (Fig. 1) indicating their lung cancer-related functions. Interestingly, all the 20 genes were discovered by CVAA indicating the power of this new method and the necessity of the comprehensively identification of DEGs.

### Early lung cancer signatures provide insights into early lung cancer diagnosis

**Fig. 2:**
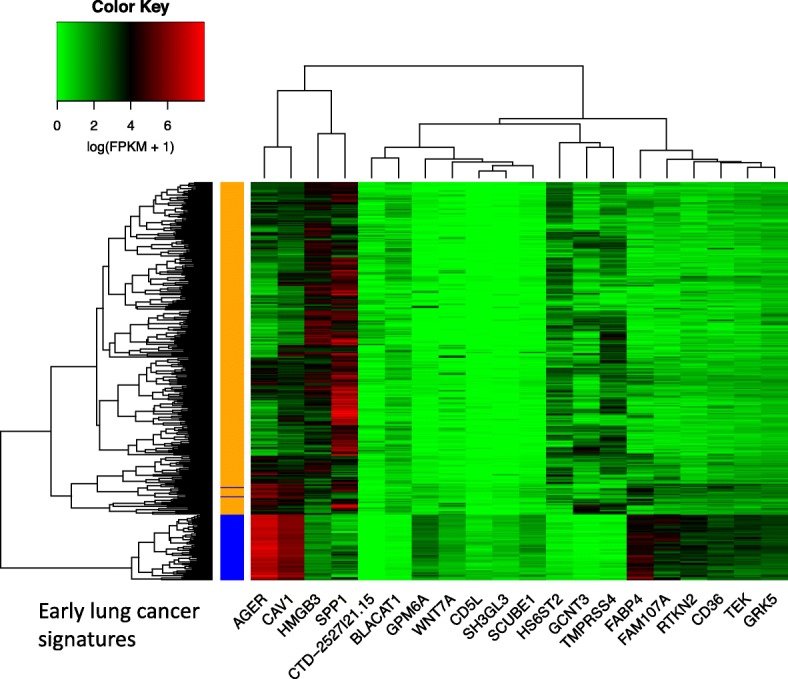
The expressions of the 20 early lung cancer signatures in TCGA lung adenocarcinoma normal (59, blue) and early (286, gold, stage I) cases

A large portion of early-stage NSCLC can be cured [2]. Lung cancer deaths are mainly caused by the distant metastases that drive cancer into late stages [2]. Early diagnosis of lung cancer is critical for patient survival and treatment. The expression patterns of our 20 early lung cancer signatures were distinct between the normal and early stage of the TCGA lung adenocarcinoma samples (Fig. 2) suggesting their potential capability for early lung cancer prediction. We next examined the effectiveness of these biomarkers by employing widely used machine learning classification algorithms.

We first applied random forest [20] for detecting the early lung cancer cases (Methods). The gene expression profile of TCGA lung adenocarcinoma dataset consisting of 59 normal and 286 early samples that reported as stage I were downloaded. The expression patterns of the signature genes of this dataset were shown in Fig. 2. The average prediction accuracy of the random forest model was 98.86% (Table 1, Method) based on the expression profiles of these signature genes.

**Table 1:** The early lung cancer prediction performances on four different datasets using random forest

Model	Assessment	TCGA	GSE68465	GSE10072	GSE89843 (Blood)
Random Forest	Accuracy	98.68%	99.51%	97.91%	64.35%
	Sensitivity	99.28%	99.95%	98.05%	78.12%
	Specificity	95.68%	92.83%	97.75%	50.59%

We then collected the second independent early lung cancer dataset: GSE68465 [21] which was generated using the microarray platform (HG-U133A). The dataset consisted of 276 early (stage IA and IB) lung cancer and 19 normal samples. Two lncRNAs (BLACAT1, CTD-2527I21.15) and three protein-coding genes (*SCUBE1, HS6ST2, RTKN2*) of the signatures were not included in this dataset. We achieved 99.51% prediction accuracy, 99.95% sensitivity, and 92.83% specificity in average for this dataset (Table 1). The third dataset (GSE10072) [22] was also microarray platform-based and contained 58 lung cancer and 49 normal cases. The patients were grouped into never, former, and current smokers by their smoking behaviors. Using the expression profile of same genes as the second dataset, we obtained 97.91% accuracy for lung cancer case prediction (sensitivity = 98.05%, specificity = 97.75%).

Blood-based liquid biopsies provide promising non-invasive cancer detection. Blood-based biomarkers have been studied and identified [23]. Based on the age-matched tumor-educated blood platelets (TEPs) early lung cancer samples (GSE89843) [23], we assessed the effectiveness of our 20 gene signatures identified from tissue samples on these TEPs data (Methods). However, the prediction accuracy is relatively lower (64.35%), (Table 1), suggesting these signatures might be tissue-specific.

**Fig. 3:**
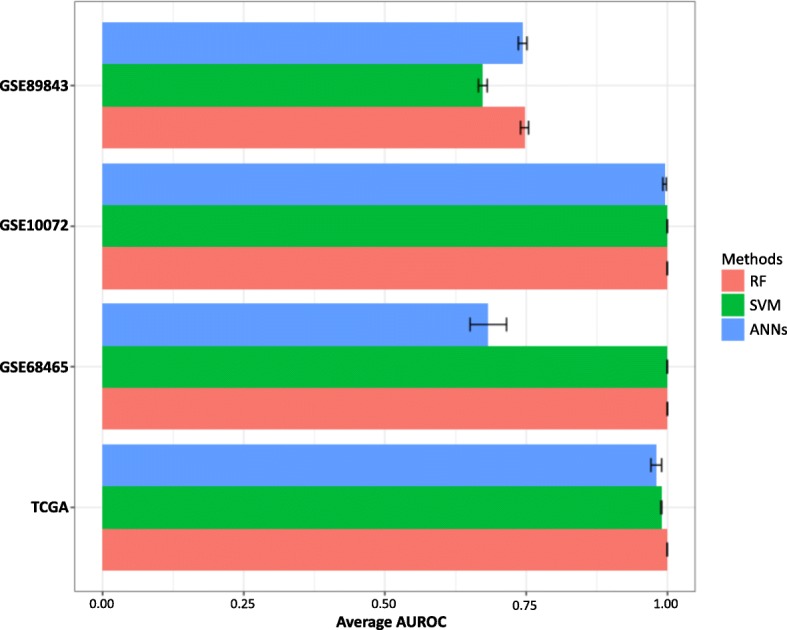
The performance assessments for early lung cancer prediction using random forest, SVM and artificial neural networks for four lung cancer datasets. The AUROC were calculated based on 100 boostrapping tests.

We further examined the prediction performances using different machine learning algorithms including random forest, SVM [24], and ANNs [25] crossing the four datasets. To comprehensively measure the robustness of our signature genes, we calculated the average area under an ROC curve (AUROC) values of each model for each dataset (Fig. 3, Additional file 6: Figure S3). All the machine learning models succeed in predicting the early lung cancer tissue samples, excepting the ANNs based model for GSE68465. GSE68465 contained unbalance samples size (19 normal vs. 276 tumor, Methods). In summary, the early lung cancer signature genes we identified showed the robustness and high accuracy for distinguishing normal and early lung cancer cases.

### The early lung cancer signature genes were highly lung cancer related

We conducted further literature search and found that majority early lung cancer signature genes we identified were reported to be highly associated with cancer progression, diagnosis, therapy, and patient overall survival. All the 18 protein-coding genes were found to be directly involved in lung cancer development suggested by previous studies (Additional file 5: Table S3). For instance, the protein-coding genes *CD36* [26] and *TMPRSS4* [27] were already identified as potential therapeutic targets of lung cancer, while *TMPRSS4* can induce cancer stem cell-like properties in lung cancer [28]. *HMGB3* and *FABP4* showed their high diagnostic and prognostic value in human NSCLC [29, 30]. *SPP1*, *AGER*, and *RTKN2* regulate the lung cancer-related pathways such as VEGF (vascular endothelial growth factor) signaling pathway and NF-kappaB [31, 32]. The loss of *WNT7A* is a major contributing factor for increased lung cancer tumorigenesis [33]. The expression level of *FAM107A* is decreased in patients with NSCLC [34], whereas the high levels of expression of *HS6ST2* are observed in lung cancer cell lines [35].

The associations of the two lncRNAs and NSCLC are not reported yet. The lncRNA BLACAT1 (Bladder Cancer Associated Transcript 1) was up-regulated in bladder cancer. BLACAT1 also affects cell proliferation, indicates a prognosis of colorectal cancer and is significantly associated with poor overall survival [36]. Our results suggested diagnostic value of BLACAT1 for NSCLC. The other lncRNA CTD-2527I21.15 is a basal-like breast cancer marker. CTD-2527I21.15 locates adjacently to FXYD3 in chromosome 19 and potentially cis-regulates its expression in cancer [37]. Moreover, our results indicated combinatory effect of these genes for early lung cancer diagnosis.

## Methods

### Data collection and processes

The raw RNA-seq data of the AIS cases (GSE52248) were downloaded. The low-quality reads were trimmed via Trimmomatic version 0.36 [38]. The human gene annotation of Ensembl was used. We applied STAR (v2.4) [39] followed by Cufflinks (v2.2.1) [40] to calculate the gene expressions. The other four independent lung cancer datasets were TCGA lung adenocarcinoma, GSE68465, and GSE10072 of which the gene expression profiles were available and GSE89843 which was a blood platelets RNA-seq library. The TCGA lung adenocarcinoma dataset was consisted of 596 samples. In this study, only the 59 normal samples and the 286 early lung cancer (stage I) samples were used for the analysis. The dataset GSE68465 was generated by microarray platform HG-U133A and collected from 6 contributing treatment institutions. The patients were around 64 years old on average and 42.3% of the patients were dead in about 4 years after the clinical report. Here, only the gene expression profiles of 19 normal samples and 276 early (stage IA and IB) lung cancer samples were used for the prediction. GSE10072 was also a microarray data and the fresh frozen lung cancer tissue samples were collected from patients with never (20), former (26), and current (28) by smoking behaviors. Additional 49 normal samples were used as control. All the samples were generated by Environment and Genetics in Lung Cancer Etiology (EAGLE). The RNA-seq data of the blood platelets of 53 early locally advanced NSCLC patients were collected from the study of GSE89843 [23]. The other 53 healthy age-matched (range from 48 to 86) samples in the same study were used as normal controls for the prediction. The gene expressions (FPKM) were calculated using the raw RNA-seq reads.

### Differentially expressed gene identification

The read counts of the genes were calculated by HTSeq-count (v0.6.1) [41]. Then, the R package edgeR was applied for differential expression analysis between the samples of various types. The threshold |log2 fold change > 1| & FDR < 0.05 was used in our study for defining significantly differentially expressed genes. The R package of the CVAA (version 0.1.0) method was obtained from the author and applied under the default setting [12]. The genes were ranked by CVAA based on the significance of the differential expression. We selected the same number of the top CVAA DEGs and the top edgeR DEGs for the further analysis. The individual sets of AIS-specific genes identified by edgeR and CVAA were combined together.

CVAA is a normalization-free and nonparametric method that identifies DEGs.

### Regulation prediction by GENIE3

GEne Network Inference with Ensemble of trees (GENIE3) calculates the regulatory relationships between genes based on the expression patterns [19]. The gene expression profile of normal and early stage of the TCGA lung adenocarcinoma sample was used. The 14 AIS-specific lncRNAs were considered as regulators while all the protein-coding genes were used as potential target genes. All the regulations between lncRNAs and protein-coding genes were ranked by the weight (Additional file 7: Figure S4) and only the regulations over the third quartile of all the weights were considered as confident regulations.

The odd ratios were calculated as:

$$ OR=\frac{P_I{R}_T/{P}_I{R}_N}{P_O{R}_T/{P}_O{R}_N} $$

Where P_I_R_T_ represents the number of the target genes of a given lncRNA that in (*I*) the ECM-receptor interaction pathway (*P*) whereas P_I_R_N_ represents the non-target genes in the pathway. P_O_R_T_ and P_O_R_N_ in the denominator stand for the number of target and non-target genes outside of (O) the pathway, respectively.

### Machine learning models for predicting the early lung cancer

Random forest allows for measuring the importance of the features, which are the genes in our study, for classification. The function of random forest cross-validation for feature selection (rfcv) was applied to reveal the best gene set for the cancer cases prediction. We used the arguments: 5-fold cross-validation, log scale, and 0.9 step which means 10% of the features were removed at each step of testing.

Then we compared classification performances of three machine learning models, random forest, SVM, and ANNs. Random Forest is an ensemble learning method that can be used for classification. The randomForest package [20] was used with 1000 trees and seed 115 for reproducibility. The e1071 is one widely used R package for performing SVM [24]. The tune function was used for detecting the best parameters of cost and gamma of SVM. The package neuralnet was used for performing the ANNs [25]. Here, we used two hidden layers with 50 and 25 neurons respectively. For each dataset, we randomly selected 2/3 of the samples as training set and the other 1/3 as testing set. Then, the average assessments of the accuracy, sensitivity, specificity, and the area under the receiver operating characteristic curve (AUROC) were calculated by running the experiment 100 times.

## Discussions

AIS cases represent the minority of lung cancer cases, however, they provide valuable information about early diagnosis and treatment of the disease. With more attention and the availability of NGS data of AIS cases, we expect more comprehensive analysis for lung cancer can be conducted.

The identification of the differentially expressed genes is critical in cancer studies. Several computational methods for differential expression analysis were developed [11, 12, 40, 42]. Most of these methods are normalized based and assume the distribution of the gene expression profile. On the other hand, the results of these differential expression analysis methods are often not consistent. Here, in addition to apply edgeR, we employed the newly developed CVAA, a normalization free and nonparametric approach for differential expression analysis. Out of 719 significant DEGs between normal and AIS cases identified by edgeR and CVAA, the overlap rate was about 50% on average (Additional file 8: Figure S5A). Moreover, less than 20% of 98 DEGs between AIS and invasive lung cancer were common genes revealed by both methods (Additional file 8: Figure S5B). Thus, the union set of the AIS-specific genes identified by edgeR and CVAA can provide a more comprehensive and robust gene set as candidate involved in lung cancer progression. Interestingly, the 20 early lung cancer gene signatures, which are the most discriminative genes in classifying normal and early cancer cases, were all identified by CVAA.

The second dataset (GSE68465) is unbalanced, which contained 19 normal samples and 276 lung cancer samples. The prediction performances of the ANNs model was poor compared with random forest and SVM for this data, suggesting performance of ANNs was impacted more by unbalanced dataset. The performance of ANNs on unbalanced data might be improved by optimizing paramters.

Tumor is highly heterogeneous and poses significant challenges in diagnosis and treatment. The gene expression profiles were different between two subtypes of the same tumor or between tissue and liquid sample types from the same patients. Our finding in this study indicted the limitation of the biomarkers that identified from tissue lung cancer samples for predicting the blood-based data.

## Conclusions

In this study, we identified the AIS-specific genes that potentially dominate the lung cancer procession from AIS into the invasive tumor. A further analysis of these specific genes in AIS revealed their essential functions and properties in diverse types of lung cancer tissues. We also identified several novel lncRNAs that were involved in lung cancer by interacting with the lung cancer-related pathways. Twenty early lung cancer signature genes were identified. A cross assessment based on diverse machine learning models and independent datasets indicated our signatures were robust for early lung cancer prediction. These signature genes were highly lung cancer-related, and the combined gene group showed the capability to improve the early lung cancer diagnosis with high accuracy.

## Additional files

Additional file 1: **Figure S1.** Gene expression comparison between normal, AIS, and invasion lung cancer cases. (PDF 334 kb)

Additional file 2: **Table S1.** List of the 107 AIS-specific genes. (XLSX 14 kb)

Additional file 3: **Table S2.** The functional annotations of the 107 AIS-specific genes. (XLSX 12 kb)

Additional file 4: **Figure S2.** The AIS-specific lncRNAs that potentially regulate the target genes in ECM-receptor interaction pathway. (PDF 576 kb)

Additional file 5: **Table S3.** List of the 20 early lung cancer signature genes and their cancer related functions. (XLSX 11 kb)

Additional file 6: **Figure S3.** An example ROC curve of three machine learning algorithms on TCGA lung adenocarcinoma dataset. The AUROC values were calculated based on one of the 100 randomly selected training and testing datasets. (PDF 49 kb)

Additional file 7: **Figure S4.** The distribution of the regulatory weights calculated by GENIE3. (PDF 173 kb)

Additional file 8: **Figure S5.** Consistency comparison between the two differential expression analysis methods. (PDF 452 kb)

## Abbreviations

AIS: Adenocarcinoma in situ
ANNs: Artificial neural networks
AUROC: Average area under an ROC curve
BLACAT1: Bladder Cancer Associated Transcript 1
CVAA: Cross-Value Association Analysis
DE: Differential expression
DEGs: Differentially expressed genes
FFPE: Formalin fixation and paraffin embedding
GENIE3: Gene Network Inference with Ensemble of trees
lncRNA: Long non-coding RNAs
NSCLC: Non-small cell lung cancer
SVM: Support vector machine
TCGA: The Cancer Genome Atlas
TEPs: Tumor-educated blood platelets
