# Supplementary figures and images for: Genomic analyses based on pulmonary adenocarcinoma in situ reveal early lung cancer signature
Source: BMC Med Genomics. 2018 Nov 20;11(Suppl 5):106. doi: 10.1186/s12920-018-0413-3 (PMC6245590; doi:10.1186/s12920-018-0413-3)

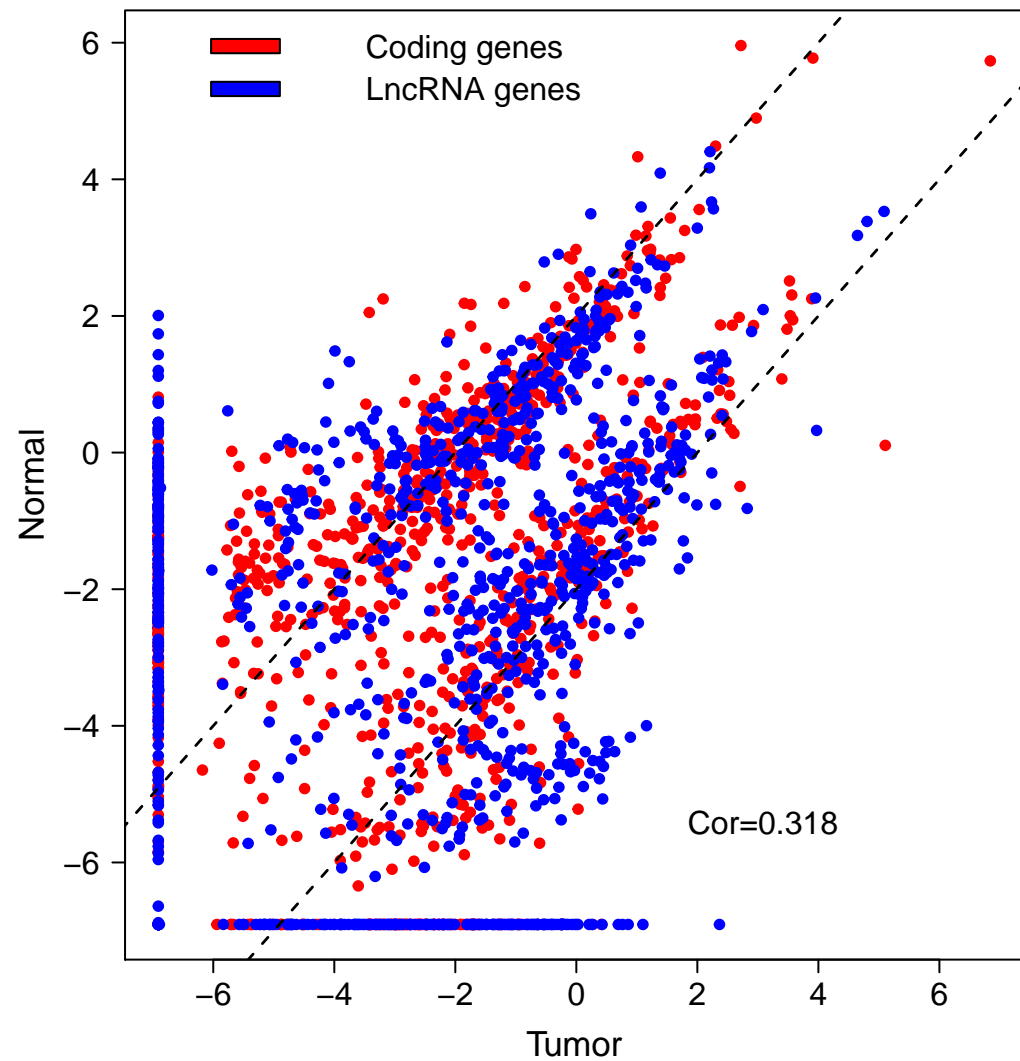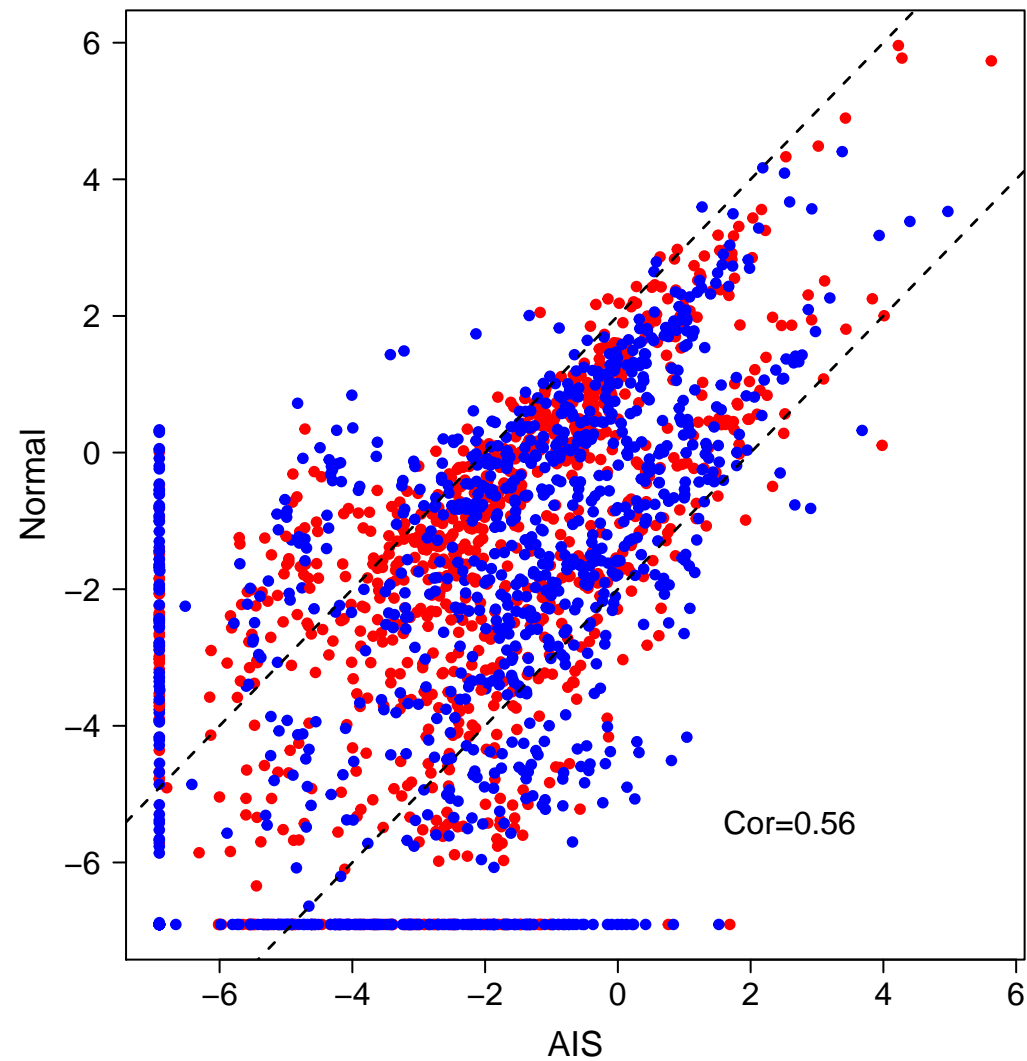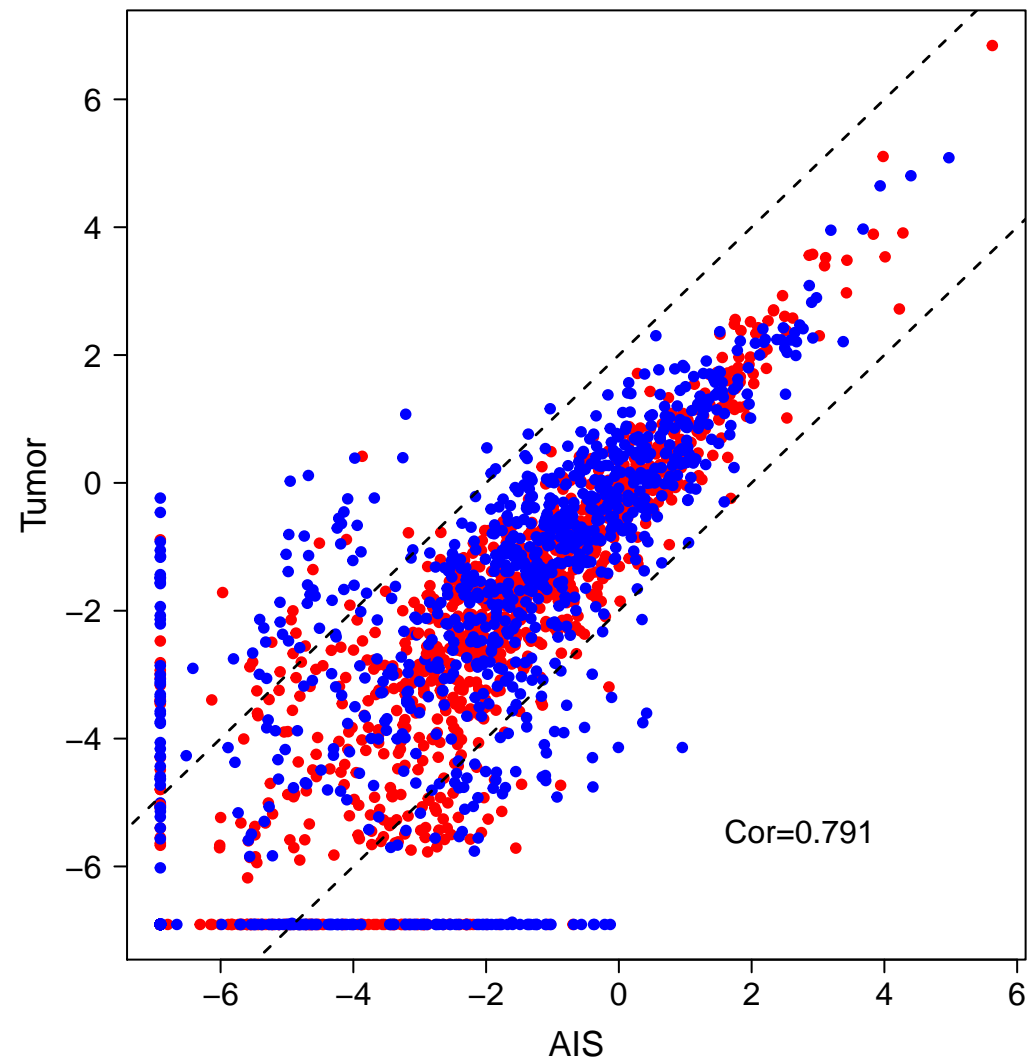

Supplement: Supplementary file 1 — Figure S1. Gene expression comparison between normal, AIS, and invasion lung cancer cases. (PDF 334 kb) [file 12920_2018_413_MOESM1_ESM.pdf]

# ECM-receptor interaction pathway regulated by lncRNAs

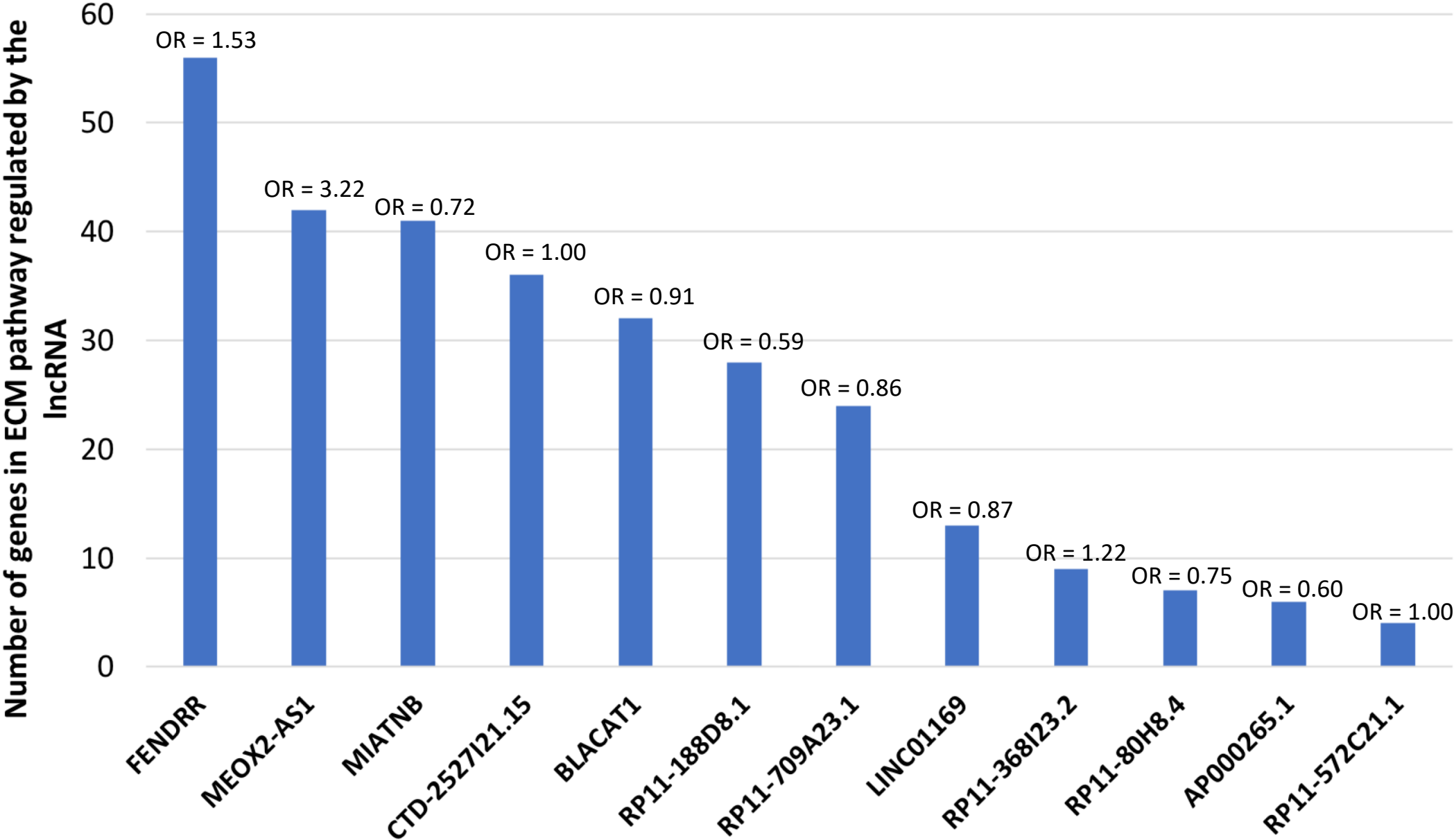

Supplement: Supplementary file 4 — Figure S2. The AIS-specific lncRNAs that potentially regulate the target genes in ECM-receptor interaction pathway. (PDF 576 kb) [file 12920_2018_413_MOESM4_ESM.pdf]

## ROC Assesment

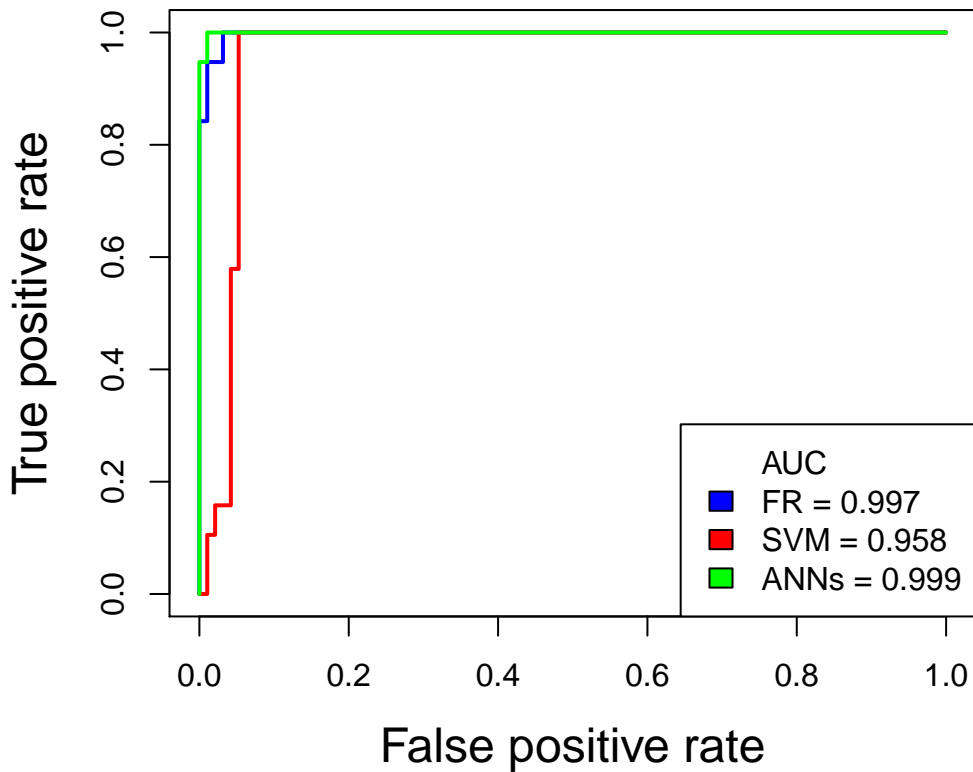

Supplement: Supplementary file 6 — Figure S3. An example ROC curve of three machine learning algorithms on TCGA lung adenocarcinoma dataset. The AUROC values were calculated based on one of the 100 randomly selected training and testing datasets. (PDF 49 kb) [file 12920_2018_413_MOESM6_ESM.pdf]

# Distribution of regulatory weights by GENIE3

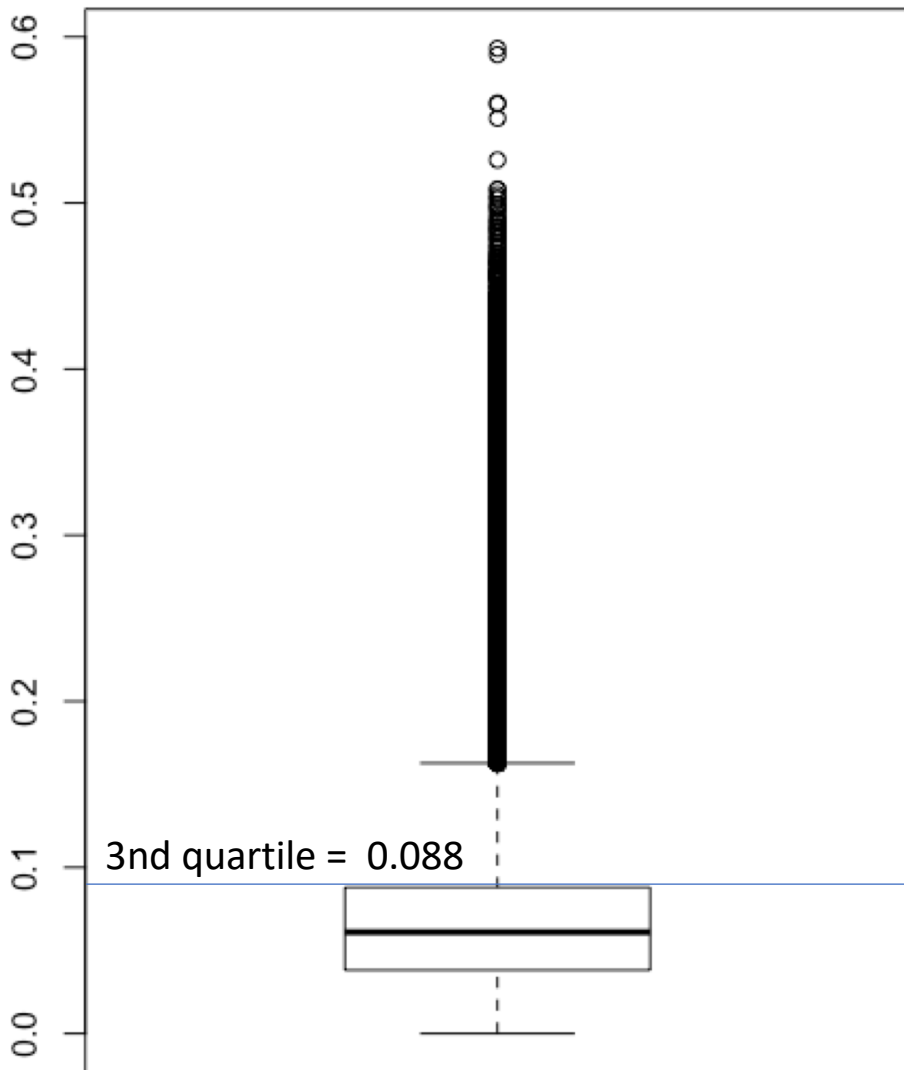

Supplement: Supplementary file 7 — Figure S4. The distribution of the regulatory weights calculated by GENIE3. (PDF 173 kb) [file 12920_2018_413_MOESM7_ESM.pdf]

A

## Common DEGs (normal vs. AIS)

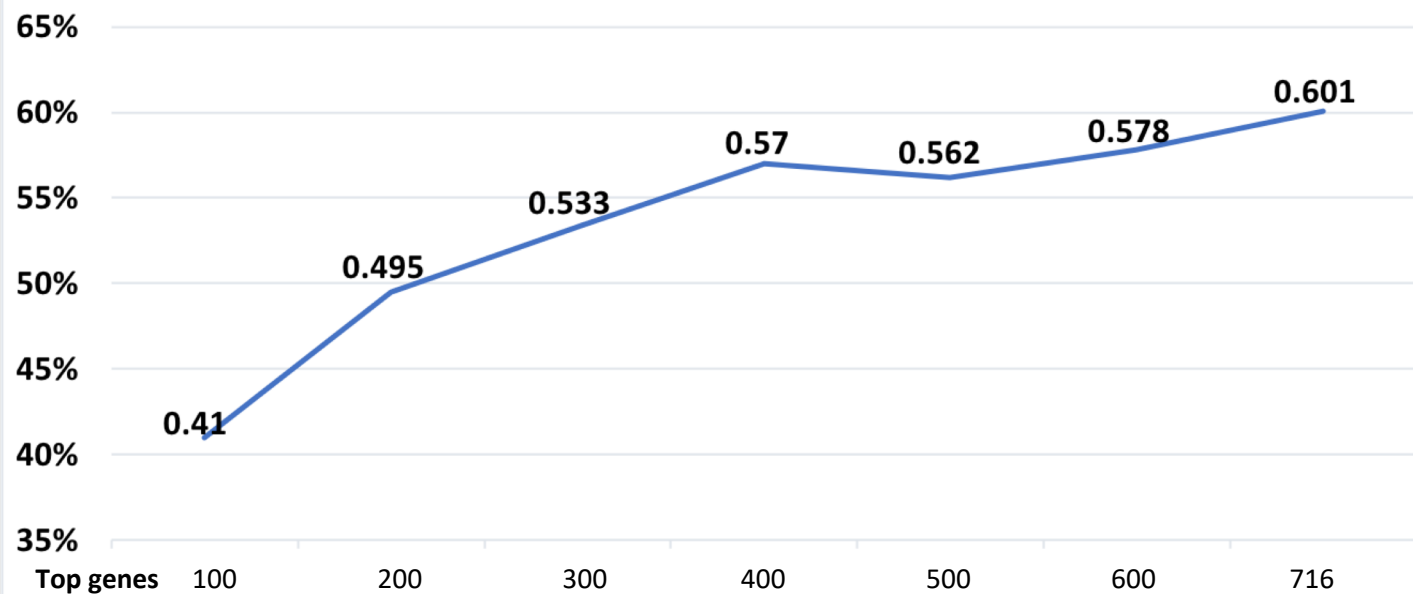

B

## Common DEGs (AIS vs. Invasive)

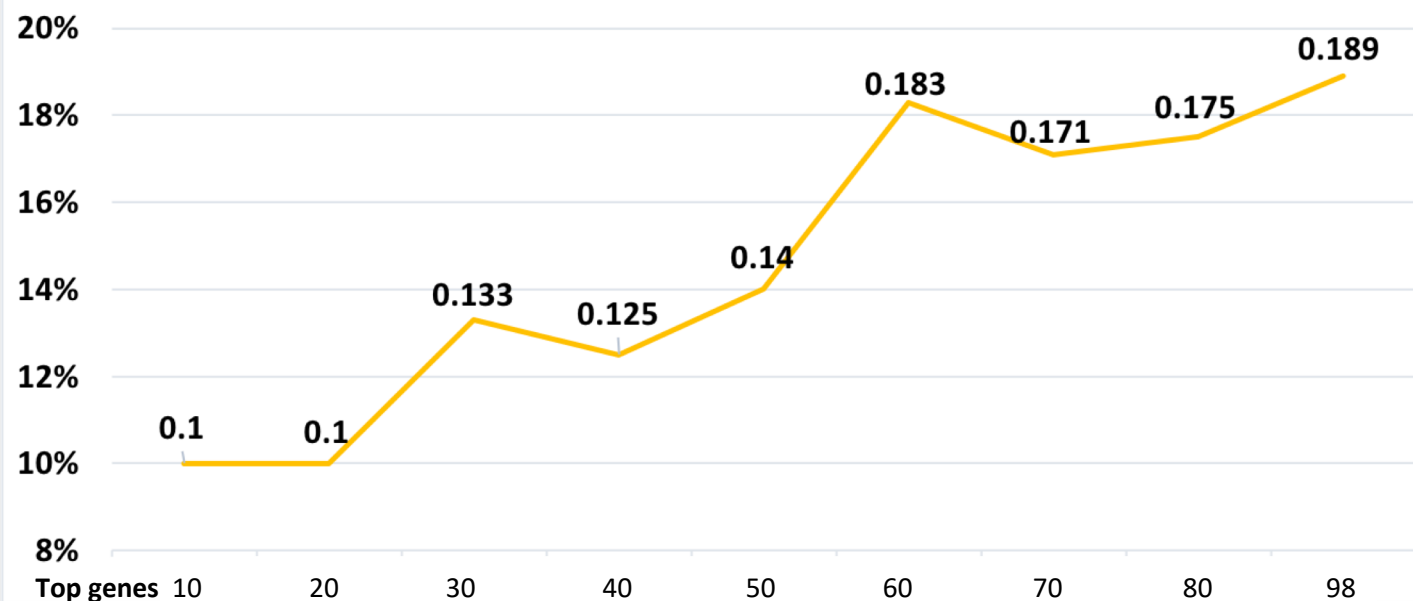

Supplement: Supplementary file 8 — Figure S5. Consistency comparison between the two differential expression analysis methods. (PDF 452 kb) [file 12920_2018_413_MOESM8_ESM.pdf]
